# Comparative evaluation of DNase-seq footprint identification strategies

**DOI:** 10.3389/fgene.2014.00278

**Published:** 2014-08-15

**Authors:** Iros Barozzi, Pranami Bora, Marco J. Morelli

**Affiliations:** ^1^Department of Experimental Oncology, European Institute of OncologyMilan, Italy; ^2^Center for Genomic Science of IIT@SEMM, Fondazione Istituto Italiano di Tecnologia (IIT)Milan, Italy

**Keywords:** DNase-seq, footprinting, gene regulatory networks, bioinformatics tools and databases, comparison of methods

## Abstract

DNase I is an enzyme preferentially cleaving DNA in highly accessible regions. Recently, Next-Generation Sequencing has been applied to DNase I assays (DNase-seq) to obtain genome-wide maps of these accessible chromatin regions. With high-depth sequencing, DNase I cleavage sites can be identified with base-pair resolution, revealing the presence of protected regions (“footprints”), corresponding to bound molecules on the DNA. Integrating footprint positions close to transcription start sites with motif analysis can reveal the presence of regulatory interactions between specific transcription factors (TFs) and genes. However, this inference heavily relies on the accuracy of the footprint call and on the sequencing depth of the DNase-seq experiment. Using ENCODE data, we comprehensively evaluate the performances of two recent footprint callers (Wellington and DNaseR) and one metric (the Footprint Occupancy Score, or FOS), and assess the consequences of different footprint calls on the reconstruction of TF-TF regulatory networks. We rate Wellington as the method of choice among those tested: not only its predictions are the best in terms of accuracy, but also the properties of the inferred networks are robust against sequencing depth.

## Introduction

DNase I treatment reveals accessible genomic regions by preferentially cleaving DNA that is not packed in heterochromatin (Cockerill, 2011). These regions, called DNase Hypersensitive Sites (DHSs), are available to the binding of transcription factors (TFs) and are therefore likely to be involved in the process of regulation of gene expression. Recently, DNase has been coupled to Next-Generation Sequencing (DNase-seq), leveraging specific computational tools (Boyle et al., 2008; Zhang et al., 2008; McCarthy and O'Callaghan, 2014). It is now possible to identify DHSs from the distribution of the aligned reads, and gain a genome-wide perspective of the structure of the open chromatin. DHSs usually span from a few hundred to a few thousand nucleotides, and their sequences often contain binding motifs for a number of TFs much larger than those that are actually bound, making it difficult to determine the exact combination of TF binding within the region in the specific experimental condition.

However, when DNase-seq is pushed to very high sequencing depths, the “footprints” of molecules bound to the DNA become appreciable as modulations of the read distribution within DHSs and can be used to determine the precise location of a binding event. This piece of information can in turn be coupled with known binding motif analysis to identify the DNA-binding protein involved in the event. The ENCODE project (Consortium, 2004; Thurman et al., 2012) combined high depth DNase-seq data together with a new metric that is sensitive to abrupt drops in the DNase signal within a DHS [the Footprint Occupancy Score (FOS)] (Neph et al., 2012c) and defined the TF-binding landscape in multiple cell lines at an unprecedented resolution. This paved the way to the reconstruction and characterization of networks of TF-TF interactions [a subset of the gene regulatory network (GRN) limited to interactions between TFs] for a large number of cell types (Neph et al., 2012b).

Footprint detection involves the recognition of a specific signature in the read density, which requires dedicated algorithms in order to be located. Pioneering approaches were proposed and applied to yeast (Hesselberth et al., 2009) and mammalian cells (Boyle et al., 2011; Pique-Regi et al., 2011). These methods were reviewed and compared by a recent publication (Piper et al., 2013), which introduced Wellington, a method for footprint detection which leverages the characteristic pattern of strand imbalance in the sequenced fragments surrounding the protein-DNA binding sites. In that study, Wellington scored best against the previously published tools. DNaseR (http://www.bioconductor.org/packages/devel/bioc/html/DNaseR.html) is another recently developed algorithm that instead utilizes the Skellam distribution to detect the same imbalance between sequencing reads on the two strands, thus representing a potential alternative to Wellington. Here, using extensive ChIP-seq data from ENCODE, we evaluate the footprint predictions obtained with DNaseR and Wellington in K562 cells and provide a detailed comparison of the performances of the two methods, also in relation to the footprints predicted by the FOS (Neph et al., 2012c). ChIP-seq (Chromatin Immuno-Precipitation followed by high-throughput sequencing) experiments are used to identify the binding sites to the DNA of a specific TF genome-wide; therefore, if ChIP-seq datasets are available, the presence of a ChIP-seq peak overlapping with a footprint can be used as a validation of the footprint itself.

Differences in the sets of predicted footprints may lead to very large differences in the regulatory interactions inferred, depending on the sequences spanned. To assess the impact of using Wellington or DNaseR on downstream analyses, we reconstructed the TF-TF network in three cell lines [K562, skeletal muscle cells (SkMC), and HepG2] with both methods, and compared the results with those in Neph et al. (2012b).

Finally, it has been shown that in a typical DNase-seq experiment, the number of footprints saturates only after reaching a very high sequencing depth (>400 millions aligned reads) (Neph et al., 2012c). Given this observation, we also evaluate how the number of footprints and the reconstructed networks depend on the read coverage by progressively down-sampling the alignment files.

To the best of our knowledge, this is the first comparison of the two most recent footprint callers (Wellington and DNaseR), relative to the original method (based on the FOS metric) proposed by ENCODE (Neph et al., 2012c) and the only assessment of the TF-TF regulatory networks predicted by sets of footprints. For these reasons, our results represent a useful resource for the field.

## Methods

Digital Genomic Footprinting (DGF) data (Hesselberth et al., 2009; Thurman et al., 2012), corresponding to DNase-seq experiments sequenced to depths high enough to detect footprints—see Hesselberth et al. (2009) and Thurman et al. (2012) for details about the experimental protocol—as well as ChIP-seq datasets for TFs in K562, HepG2, and SkMC cell lines were downloaded from the repository where the ENCODE data are stored, namely the golden path of the UCSC genome browser (Fujita et al., 2011). Genome-wide TF-binding maps were generated using FIMO (Grant et al., 2011) and published PWMs (Wei et al., 2010; Kulakovskiy et al., 2013).

Genomic coordinates of the footprints published in Neph et al. (2012c) in K562, HepG2, and SkMC cell lines, based on the same DGF data and obtained with the FOS metric, were downloaded from ftp://ftp.ebi.ac.uk/pub/databases/ensembl/encode/integration_data_jan2011/byDataType/footprints/. Thresholds on footprint calls for DnaseR (http://www.bioconductor.org/packages/devel/bioc/html/DNaseR.html) and Wellington v. 0.1.0 (Piper et al., 2013) were chosen in order to obtain a number of footprints comparable to Neph et al. (2012c). Only footprints contained in DHSs were considered. All the datasets used are collected in Table S1.

Network reconstruction was performed according to the procedure described in Neph et al. (2012b). For each TF, a window of 10 kbps centered on the RefSeq TSSs was scanned for matches of PWMs in Transfac (Matys et al., 2006) using FIMO (Grant et al., 2011) and overlapped with footprints using BEDOPS (Neph et al., 2012a).

Receiver-Operator Characteristics (ROCs) and Areas Under the Curve (AUCs) were generated with the ROCR package (Sing et al., 2005). The igraph R package (Csardi and Nepusz, 2006) was used to compute large-scale properties of the inferred networks and to generate random networks.

Further details are provided in the Extended Methods in Supplementary Material.

## Results

### Comparison of footprint callers

Following Piper et al. (2013), we considered the datasets in K562 cell line from ENCODE (all the datasets used for this paper are listed in the Table S1), and we compared the predictions of TF binding by combining known motifs and footprints called either with DNaseR or Wellington. Besides, we also considered the set of footprints identified according to the FOS in the context of the ENCODE effort (Neph et al., 2012c) in the same cell line. DNaseR consistently identified more footprints than Wellington at comparable stringency levels (Figure S1). We tuned the parameters (see Extended Methods in Supplementary Material) of both approaches to obtain a number of footprints in the same order of magnitude (DNaseR: 1,075,979; Wellington: 1,833,281), which was also comparable to the number reported by Neph et al. (498,683) (Neph et al., 2012c). We only considered footprints within DHSs (as available in the ENCODE repository): while Wellington requires the genomic coordinates of the DHSs, DNaseR runs genome-wide; to have directly comparable results, we considered only the regions corresponding to the DHSs also for the results coming from DNaseR. We interpreted a footprint overlapping with one of the known binding motifs of a specific TF as a prediction for an actual binding event for the TF (see Extended Methods for details in Supplementary Material).

**Figure 1:**
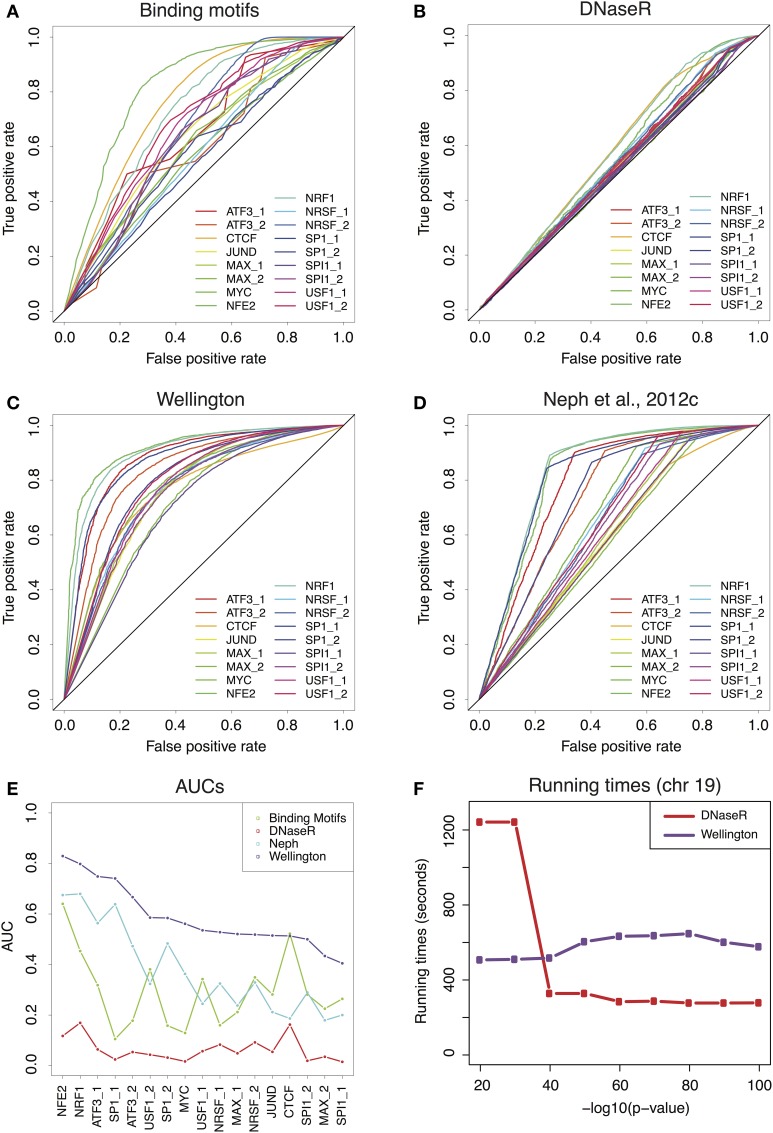
**(A)** Receiver-Operator Characteristic (ROC) curves for the predictions provided by the binding motifs alone. **(B–D)** ROCs for the sets of footprints obtained by DNaseR, Wellington and for the set used in Neph et al. (2012c). **(E)** Area Under the Curve (AUC) corresponding to the ROCs of **(A–D)** Wellington scores consistently better than all the other methods. **(F)** Running times for DNaseR and Wellington on chromosome 19, for different significance thresholds.

We extracted 17 binding patterns from ChIP-seq experiments in K562 human cells from the ENCODE repository, corresponding to 11 TFs (Piper et al., 2013). We then used the genomic coordinates of the ChIP-enriched regions to validate the footprint predictions. We computed Receiver-Operator Characteristic curves (ROCs) for the predictions generated by binding motifs alone (Figure 1A), and for the three sets of footprints described above (Figures 1B–D). The global performances of the methods are summarized by the AUC of each of the ROCs, displayed in Figure 1E. Irrespectively of the considered TF, the method with the highest predictive power is Wellington. Nevertheless, it must be noted that the AUC calculated using the FOS score (Neph et al., 2012c) might be underestimated, as we could not perform a more permissive footprint call, because the required software was not released by the authors. Remarkably, overlapping motifs with DHS coordinates without considering footprints already provides a rather good prediction of the TF binding patterns, which for some TFs is in line (USF, NRSF, SPI1, MAX, JUND) or better (CTCF) than the footprints calculated in Neph et al. (2012c); conversely, DNaseR performs systematically worse than the other methods, indicating that the majority of the DNaseR footprints correspond to genomic locations where the TF is not bound. Besides, the sets of footprints obtained by DNaseR for different significance thresholds did not reduce to a simple inclusion of weaker and weaker signals but rather introduce new elements (see Figures S2, S3); on the other hand, Wellington showed the expected behavior. These observations support the idea that the method implemented by Wellington provides a better detection of the footprint signal in DNase-seq data.

Finally, we benchmarked the running times (see Extended Methods for details in Supplementary Material) of Wellington and DNaseR on chromosome 19 for several significance thresholds (Figure 1F): while Wellington consistently ran at approximately the same speed, DNaseR was remarkably slower for permissive calls.

### Robustness and characteristics of the inferred networks

After evaluating the performance of the footprint callers, we assessed the impact of different sets of footprints on downstream analyses. In particular, we reconstructed the network of TF-TF interactions following the protocol described in Neph et al. (2012b) (see Extended Methods for a detailed description in Supplementary Material). We repeated the procedure for DGF data from (1) the K562 cell line (myelogenous leukemia) used in the previous section, (2) the SkMC cell line and (3) the HepG2 (liver hepatocellular carcinoma) cell line. For each of these three cell lines, we obtained the TF-TF network using the sets of footprints coming from the three different methodologies described above (Neph, DNaseR, Wellington). Moreover, we evaluated the impact of sequencing depth on the same analysis by running Wellington on progressively down-sampled alignment files for the three cell lines and reconstructing the corresponding TF-TF networks. We chose to use the SkMC cell line because it has the highest depth of sequencing among the DNase-seq experiments performed by the ENCODE Consortium. In this dataset, the number of footprints is well saturated (Figure S4), allowing us to properly evaluate the effect of the down-sampling starting from the whole set of footprints.

**Figure 2:**
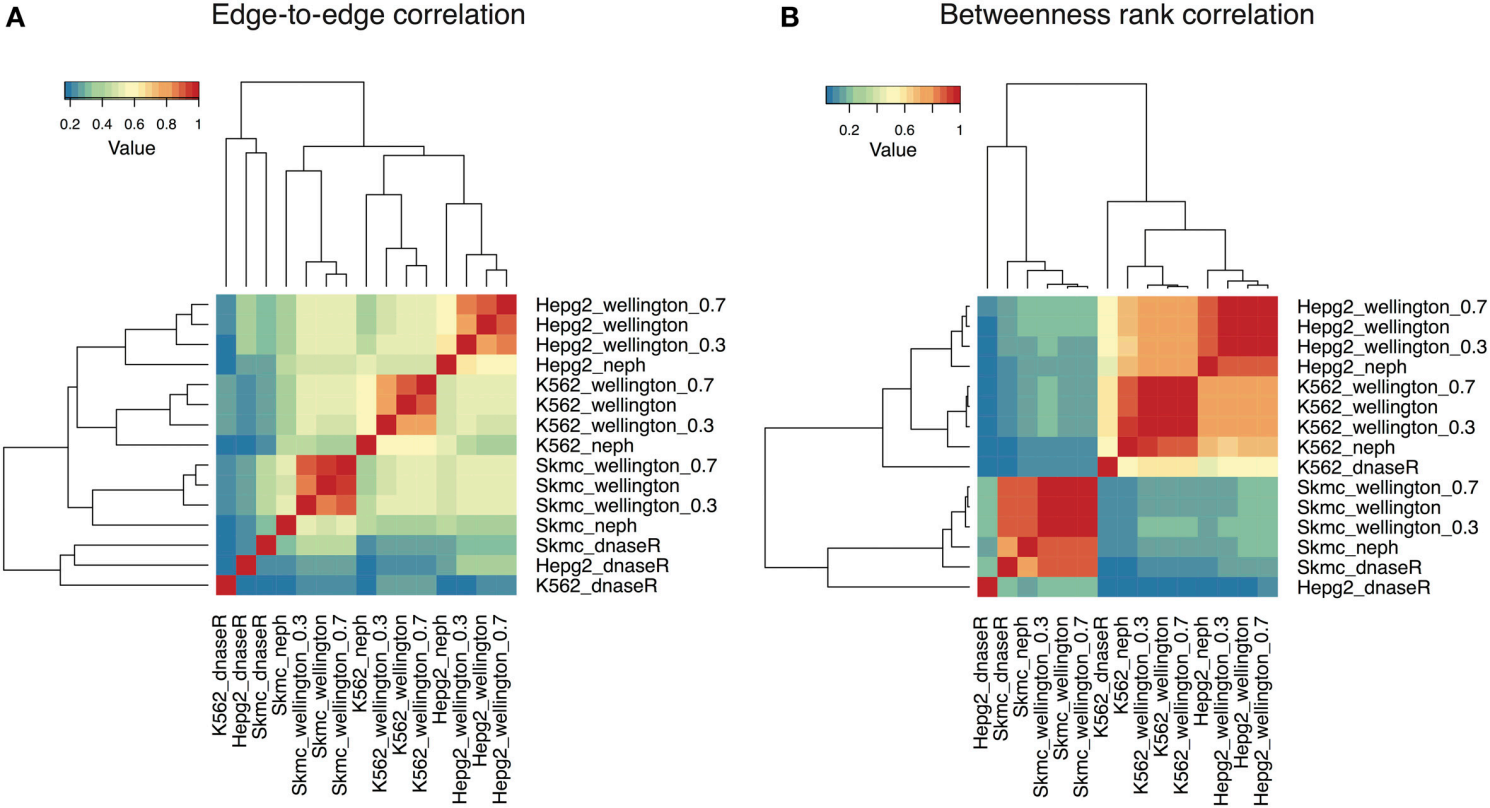
**Heatmaps summarizing the comparison among the TF-TF networks reconstructed with the sets of footprints obtained with DNaseR, Neph, Wellington in three different cell lines (K562, SkMC, HepG2)**. Networks obtained by running Wellington on 30 and 70% subsamples of aligned reads are also included. **(A)** Edge-to-edge correlation: DNaseR networks cluster separately; networks obtained with Wellington and Neph separate according to the cell type of origin. **(B)** The rank correlation of the betweenness centrality (a measure quantifying how many times a node is present in the shortest paths between two nodes) for the different networks show a comparable pattern, except that in this case K562 and HepG2 networks show much higher positive correlation between each other as compared to SkMC.

We first compared the networks by counting how often a specific edge is present between each pair of nodes: Figure 2A shows a heatmap displaying the edge-to-edge correlation between all pairs of samples. While the networks obtained with DNaseR cluster together irrespectively of the cell type they correspond to, the networks generated with the footprints computed by Wellington or obtained from Neph et al. (2012c) separate into three different clusters corresponding to the cell types considered. Notably, the networks obtained with Wellington with decreasing sequencing depth are all very similar, indicating that most of the weak footprints do not correspond to interactions between TFs with annotated binding preferences, which are in turn detectable at sub-optimal sequencing depths (Figure S4 shows that the down-sampling affects the set of footprints generating edges in the TF-TF interaction network remarkably less than the others).

To characterize the networks beyond basic local properties, we computed the betweenness centrality of each node, that is, the number of times a node acts as a bridge along the shortest path between two other nodes (Newman, 2010), and their rank correlations (Figure 2B). With the exception of the networks reconstructed with DNaseR, the three cell lines still cluster separately yet the inter-cell lines correlations are now significantly increased between K562 and HepG2, and less pronounced when compared with the SkMC. In other words, the nodes in K562 and HepG2 cells have a much more similar position within the network than SkMC cells, while the fraction of conserved edges is similar: even if two networks are locally different, the global properties of two TF-TF networks inferred in different cell lines can indeed be remarkably similar. As for the presence of shared edges, the betweenness centrality of the networks is robust against down-sampling the alignment files.

It has been previously observed (Deplancke et al., 2006) that the connections of some GRNs have a scale-free structure (characterized by presence of a few hubs, nodes which are extremely highly connected, and a large number of poorly-connected vertices, reviewed in Barabasi, 2009). Here, we concentrated on TF-TF networks, i.e., we pruned all the edges connecting a TF to target genes that are not regulating the expression of other genes. As a result, a large number of nodes with low degree are removed, and the degree distribution becomes non-monotonic and unimodal. However, the tail of this distribution decays much more slowly than the exponential tail of a random (Erdös-Rényi) network with the same number of nodes and edges, indicating that a large number of nodes have many more connections than would be expected by chance (Figure S5). Moreover, the TF-TF networks we obtained fall in the small world category—as defined by Watts and Strogatz (1998)—as they display an average path length (the shortest distance between any two nodes) comparable to that of a random network, but a consistently higher clustering coefficient (a measure of how nodes tend to cluster together), as displayed in Figure S6. These observations are consistent across the TF-TF networks obtained with the three methods (Neph, Wellington, DNaseR) under comparison.

## Discussion

We performed a systematic comparison of two state-of-the art footprint callers and one recently-introduced metric to identify footprints in DNase-seq experiments, by validating their performances using ChIP-seq data from the ENCODE project and evaluating their impact on a footprint-based reconstruction of the TF-TF regulatory network. Our results show that (1) Wellington is the method displaying the best performance and (2) network reconstruction starting from footprints called by Wellington or using the FOS approach from Neph et al. (2012b) allows a better separation of cell types with respect to DNaseR. Moreover, the networks reconstructed by Wellington are robust against the sequencing depth of the DNase-seq experiment, being not heavily dependent on the number of footprints obtained.

Recently, a tool called PIQ (Protein Interaction Quantitation) (Sherwood et al., 2014) has been proposed to identify TF binding sites and corresponding changes in chromatin structure through the detection of consistent shape patterns in the DNase sequencing profiles, with simultaneous weighting of the sequence information. However, PIQ is not a tool designed to detect footprints but rather to directly integrate DNase-seq data with known binding motifs for TFs, and we therefore decided against including it in this comparison.

It has been previously shown that the detection of footprints is related to the depth of sequencing of the DNase-seq experiment (Neph et al., 2012c). However, while down-sampling the number of reads resulted in a substantial drop in the number of footprints identified by Wellington, the local and global properties of the inferred TF-TF networks were maintained. This observation suggests that the weak footprints lost when the signal in the alignment files is less sharp are likely to be noise, or to correspond to interactions either with molecules other than TFs, or to TFs without a known binding preference. While this limitation seems not to affect the overall characteristics of the TF-TF network, it cannot be excluded that other properties do not display the same degree of robustness against sequencing depth.

### Conflict of interest statement

The authors declare that the research was conducted in the absence of any commercial or financial relationships that could be construed as a potential conflict of interest.

## Supplementary material

Table S1 **Complete list of datasets used in this study**.

Figure S1 **The number of footprints is shown as a function of the applied thresholds on statistical significance**.

Figure S2 **Genome browser screenshot showing a genomic region along with footprint calls at different *p*-values [−10^*^log10(p)]**.

Figure S3 **Each set of footprints has been compared to the set of footprints obtained at a lower *p*-value; the fraction of overlap is shown as a function of the *p*-value [−10^*^log10(p)]**.

Figure S4 **The total number of footprints and the number of footprints generating an edge in the TF-TF interaction network are shown as a function of the sampled reads**.

Figure S5 **Degree distribution for TF-TF networks (solid lines) and corresponding random networks (dashed lines)**.

Figure S6 **Average path length and clustering coefficient for TF-TF networks (black lines) and corresponding random networks (red lines)**.
