# Corrigendum: Comparative evaluation of DNase-seq footprint identification strategies

**DOI:** 10.3389/fgene.2014.00320

**Published:** 2014-09-19

**Authors:** Iros Barozzi, Pranami Bora, Marco J. Morelli

**Affiliations:** ^1^Department of Experimental Oncology, European Institute of OncologyMilan, Italy; ^2^Center for Genomic Science of IIT@SEMM, Fondazione Istituto Italiano di Tecnologia (IIT)Milan, Italy

**Keywords:** DNase-seq, footprinting, gene regulatory networks, bioinformatics tools and databases, comparison of methods

Figure 1 of the article Comparative evaluation of DNase-seq footprint identification strategies, by Barozzi et al. (2014) contained a minor mistake, which we correct here. In panel E, the y axis ranges from 0.5 to 1 and not from 0 to 1 as indicated in the original figure. We resubmit a corrected version of Figure 1.

**Figure 1:**
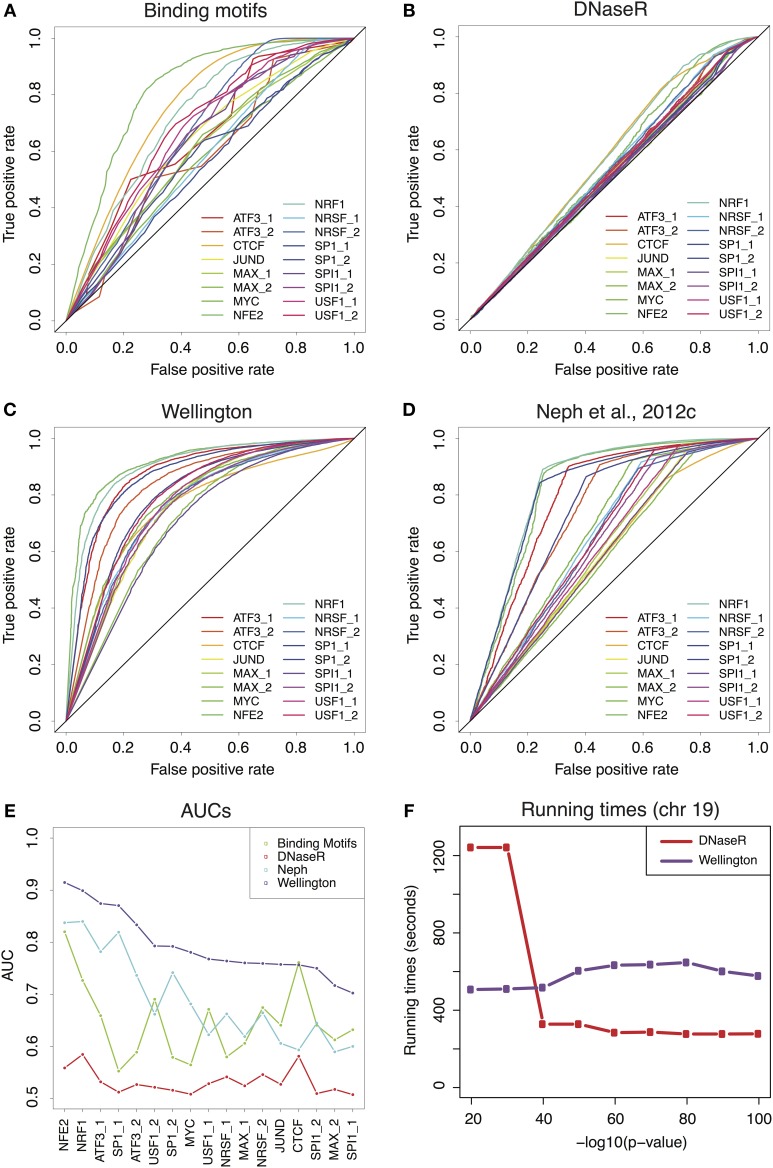
**(A)** Receiver-Operator Characteristic (ROC) curves for the predictions provided by the binding motifs alone. **(B–D)** ROCs for the sets of footprints obtained by DNaseR, Wellington and for the set used in Neph et al.(2012c). **(E)** Area Under the Curve (AUC) corresponding to the ROCs of **(A–D)** Wellington scores consistently better than all theother methods. **(F)** Running times for DNaseR and Wellington on chromosome19, for different significance thresholds.

## Conflict of interest statement

The authors declare that the research was conducted in the absence of any commercial or financial relationships that could be construed as a potential conflict of interest.
