# Supplementary material for: Comparative evaluation of DNase-seq footprint identification strategies
Source: Front Genet. 2014 Aug 15;5:278. doi: 10.3389/fgene.2014.00278 (PMC4133688; doi:10.3389/fgene.2014.00278)
Supplement: Table S1 — Complete list of datasets used in this study. [file DataSheet1.PDF]

Extended Methods

DNaseI-seq datasets

Digital Genomic Footprinting (DGF) data (Thurman et al., 2012) for K562, HepG2 and Skmc cell lines were downloaded from the UCSC genome browser (Fujita et al., 2011) golden path (hg19 release of the human genome). Data include alignments (bam format) as well as DHSs (DNase Hypersensitive Sites, broadPeak format) calls performed with the HotSpot algorithm (John et al., 2011).

Transcription factor datasets

ChIP-seq datasets for the following Transcription Factors (TFs): ATF3, CTCF, JUND, MAX, MYC, NFE2, NRF1, NRSF, SP1, SPI1, USF were downloaded from the UCSC genome browser (Fujita et al., 2011) golden path (hg19 release of the human genome). Using published binding preferences, a genome-wide map was generated for each TF. High-quality Position Weight Matrices (PWMs) were retrieved from the literature (SPI1: Wei et al., 2010; the remaining factors: Kulakovskiy et al., 2013). The human genome (hg19) was scanned using FIMO (Grant et al., 2011) (version included in Meme 4.6.1). After computing a log-likelihood ratio score relative to each genomic position, FIMO computes the corresponding *p*-values. Only positions with a *p*-value equal or lower than 1e-4 were kept.

Footprints detection

Genomic coordinates (hg19) of the published footprints (Neph et al., 2012 (1)) in K562, HepG2 and SkMC cell lines were downloaded from [1] (hereafter referred to as *neph* footprints).

DNaseR is available as a Bioconductor package [2]. Analyses were performed using R version 3.0.2, one chromosome at a time, and footprints collected. In order to get a number of footprints comparable to *neph*, DNaseR was run with a *p*-value threshold of 1e-4. The remaining arguments of the *footprints* function were set as follows: *width* = c(4,60), *N* = 2e6, *correction* = "BH".

Wellington v. 0.1.0 (Piper et al., 2013) is available as a package for Python [3]. Analyses were run using Python version 2.7.3 using default parameters except for: -fdr 0.01 -pv -2. This last threshold, corresponding to a *p*-value = $10^{-0.2} \sim 0.6$, was chosen in order to obtain a number of footprints comparable to *neph*. Thresholding on FDR only is a viable alternative with version 0.1.6 or higher.

For the next steps of the analysis only the footprints contained in the DHSs of the matched cell line were considered.

Benchmarks for running times were performed on an Intel(R) Xeon(R) X5670 CPU (2.93GHz) using chr19 in K562 cells.

Sub-sampling of DGF data

Random sub-sampling of sequenced reads was performed separately for each of the three datasets considered. Starting from the bam files, *samtools view* was run with the –s parameter ranging from 0.1 to 0.9 (10% to 90% down-sampling, samtools version 0.1.19, Li et al., 2009).

Reconstruction of regulatory networks

The list of TFs genes was extracted from the supplementary material of Neph et al. 2012 (2), and networks reconstructed according to the procedure applied in the same publication. In the final networks, each node stands for a TF and each directed edge corresponds to a regulatory interaction. Each one of these interactions represents a binding event of a TF to the TSS-proximal genomic region of another TF (or the considered TF itself), inferred by overlapping the TF-binding maps (predicted using only the published binding preferences for the TFs) with the footprints calls (Neph et al. 2012 (2)).

More specifically, a window of 10 kbps centered on the RefSeq TSSs was used. The table of RefSeq genes was downloaded from the UCSC Table Browser (Fujita el al., 2011) October 10th, 2012. For each TF, a TF-binding map was generated using FIMO (Grant et al., 2011) as described in the paragraph "Transcription factor datasets". PWMs were extracted from Transfac (Release 2013.2, Volker et al., 2006). It should be noted that often more than one PWM is assigned to a TF. In these cases, predicted binding sites from multiple PWM were combined before reconstructing the regulatory network. Overlaps among maps of TF-binding (obtained with FIMO) and footprints were assessed using BEDOPS (Neph et al., 2013 (3)), and a minimum of 3 overlapping base pairs between the motif and the footprint was required. Even though the parameters of the three different footprints detection methods (*neph*, dnaseR, Wellington) were tuned to result in a similar number of regions, for each of the three cell lines considered, the lists were further reduced to the size of the smallest, keeping only the most significant footprints.

Generation of ROC curves

Receiver-Operator Characteristics (ROCs) and Areas Under the Curve (AUCs) were generated with the ROCR package (Sing et al., 2005 [4]) and R version 3.0.2. For each TF and each footprint prediction method (*neph*, DNaseR, Wellington), the regions contained in the TF-binding map described in the paragraph "Transcription factor datasets" were intersected with the footprint set. Regions with no overlap with a footprint were assigned a fictious footprint with a *p*-value logarithmically distributed in the range $]p_{max}, 1]$, where p_{max} is the highest *p*-value in the corresponding footprint set. The resulting set was intersected with the enriched regions in the corresponding ChIP-seq experiment, to obtain then the following categories, similarly to (Piper et al., 2013): TP (True Positive) = region in the TF-binding map overlapping a footprint and falling within a ChIP-seq peak; FP (False Positive) = region in the TF-binding map overlapping a footprint and not falling within a ChIP-seq peak; FN (False Negative) = region in the TF-binding map not predicted by a footprint and falling within a ChIP-seq peak; TN (True Negative) = region in the TF-binding map not predicted by a footprint and not falling within a ChIP-seq peak.

Network analysis

The igraph R package (Csardi et al., 2006 [5]) and R version 3.0.2 were used to compute large-scale properties of the inferred networks (degree, betweenness centrality, clustering) and to generate random (Erdős-Rényi) networks with the same number of nodes and edges.

Links

1. ftp://ftp.ebi.ac.uk/pub/databases/ensembl/encode/integration_data_jan2011/byDataType/footprints/
2. http://www.bioconductor.org/packages/devel/bioc/html/DNaseR.html
3. http://pythonhosted.org/pyDNase/
4. http://rocr.bioinf.mpi-sb.mpg.de/
5. http://igraph.org

References

- RE Thurman, E Rynes, R Humbert, J Vierstra, MT Maurano, E Haugen, et al. The accessible chromatin landscape of the human genome. Nature 489 (7414), 75-82
- Sam John, Peter J Sabo, Robert E Thurman, Myong-Hee Sung, Simon C Biddie, Thomas A Johnson, Gordon L Hager, John A Stamatoyannopoulos, Chromatin accessibility pre-determines glucocorticoid receptor binding patterns. Nature Genetics, 43, 264-268 (2011).
- Neph, Shane, et al. "An expansive human regulatory lexicon encoded in transcription factor footprints." Nature 489.7414 (2012): 83-90.
- S Neph, AB Stergachis, A Reynolds, R Sandstrom, E Borenstein, et al. Circuitry and dynamics of human transcription factor regulatory networks. Cell 150 (6), 1274-1286. (2012)
- Piper, Jason, et al. "Wellington: a novel method for the accurate identification of digital genomic footprints from DNase-seq data." Nucleic acids research 41.21 (2013): e201-e201.
- Pauline A Fujita, Brooke Rhead, Ann S Zweig, Angie S Hinrichs, Donna Karolchik, Melissa S Cline, Mary Goldman, Galt P Barber, Hiram Clawson, Antonio Coelho, et al. The UCSC genome browser database: update 2011. Nucleic acids research, 39(suppl 1):D876–D882, 2011.
- Charles E Grant, Timothy L Bailey, and William Stafford Noble. FIMO: scanning for occurrences of a given motif. Bioinformatics, 27(7):1017–1018, 2011.
- Gong-Hong Wei, Gwenael Badis, Michael F Berger, Teemu Kivioja, Kimmo Palin, Martin Enge, Martin Bonke, Arttu Jolma, Markku Varjosalo, Andrew R Gehrke, et al. Genome-wide analysis of ETS-family DNA-binding in vitro and in vivo. The EMBO journal, 29(13):2147–2160, 2010.
- Kulakovskiy, Ivan V., et al. "HOCOMOCO: a comprehensive collection of human transcription factor binding sites models." Nucleic acids research 41.D1 (2013): D195-D202.
- Li, Heng, et al. "The sequence alignment/map format and SAMtools." Bioinformatics 25.16 (2009): 2078-2079.
- Matys, Volker, et al. "TRANSFAC® and its module TRANSCompel®: transcriptional gene regulation in eukaryotes." Nucleic acids research 34.suppl 1 (2006): D108-D110.
- Shane Neph, M. Scott Kuehn, Alex P. Reynolds, et al. BEDOPS: high-performance genomic feature operations. Bioinformatics (2012) 28 (14): 1919-1920.

- Tobias Sing, Oliver Sander, Niko Beerenwinkel, Thomas Lengauer: ROCR: visualizing classifier performance in R, Bioinformatics 21(20):3940-3941 (2005).
- Csardi, Nepusz: The igraph software package for complex network research, InterJournal, Complex Systems (2006): 1695.

Supplementary Table 1

Cell line	Data type	File Name	TF
K562	Aligned reads	wgEncodeUwDgfK562Aln.bam	
K562	DHS coordinates	wgEncodeUwDgfK562Hotspots.broadPeak	
HepG2	Aligned reads	wgEncodeUwDgfHepg2Aln.bam	
HepG2	DHS coordinates	wgEncodeUwDgfHepg2Hotspots.broadPeak	
SkMC	Aligned reads	wgEncodeUwDgfSkmcAln.bam	
SkMC	DHS coordinates	wgEncodeUwDgfSkmcHotspots.broadPeak	
K562	Peak coordinates	wgEncodeHaibTfbsK562Atf3V0416101PkRep1.broadPeak	ATF3_1
K562	Peak coordinates	wgEncodeHaibTfbsK562Atf3V0416101PkRep2.broadPeak	ATF3_2
K562	Peak coordinates	wgEncodeHaibTfbsK562MaxV0416102PkRep1.broadPeak	MAX_1
K562	Peak coordinates	wgEncodeHaibTfbsK562MaxV0416102PkRep2.broadPeak	MAX_2
K562	Peak coordinates	wgEncodeHaibTfbsK562NrsfV0416102PkRep1.broadPeak	NRSF_1
K562	Peak coordinates	wgEncodeHaibTfbsK562NrsfV0416102PkRep2.broadPeak	NRSF_2
K562	Peak coordinates	wgEncodeHaibTfbsK562Pu1Pcr1xPkRep1.broadPeak	SPI1_1
K562	Peak coordinates	wgEncodeHaibTfbsK562Pu1Pcr1xPkRep2.broadPeak	SPI1_2
K562	Peak coordinates	wgEncodeHaibTfbsK562Sp1Pcr1xPkRep1.broadPeak	SP1_1
K562	Peak coordinates	wgEncodeHaibTfbsK562Sp1Pcr1xPkRep2.broadPeak	SP1_2
K562	Peak coordinates	wgEncodeHaibTfbsK562Usf1V0416101PkRep1.broadPeak	USF_1
K562	Peak coordinates	wgEncodeHaibTfbsK562Usf1V0416101PkRep2.broadPeak	USF_2
K562	Peak coordinates	wgEncodeOpenChromChipK562CtcfPk.narrowPeak	CTCF
K562	Peak coordinates	wgEncodeSydhTfbsK562CmycStdPk.narrowPeak	MYC
K562	Peak coordinates	wgEncodeSydhTfbsK562Nfe2StdPk.narrowPeak	NFE2
K562	Peak coordinates	wgEncodeSydhTfbsK562Nrf1IggrabPk.narrowPeak	NRF1
K562	Peak coordinates	wgEncodeUchicagoTfbsK562EjundControlPk.narrowPeak	JUND

Supplementary Table 1. List of the ENCODE data used in this study.

Supplementary Figure 1

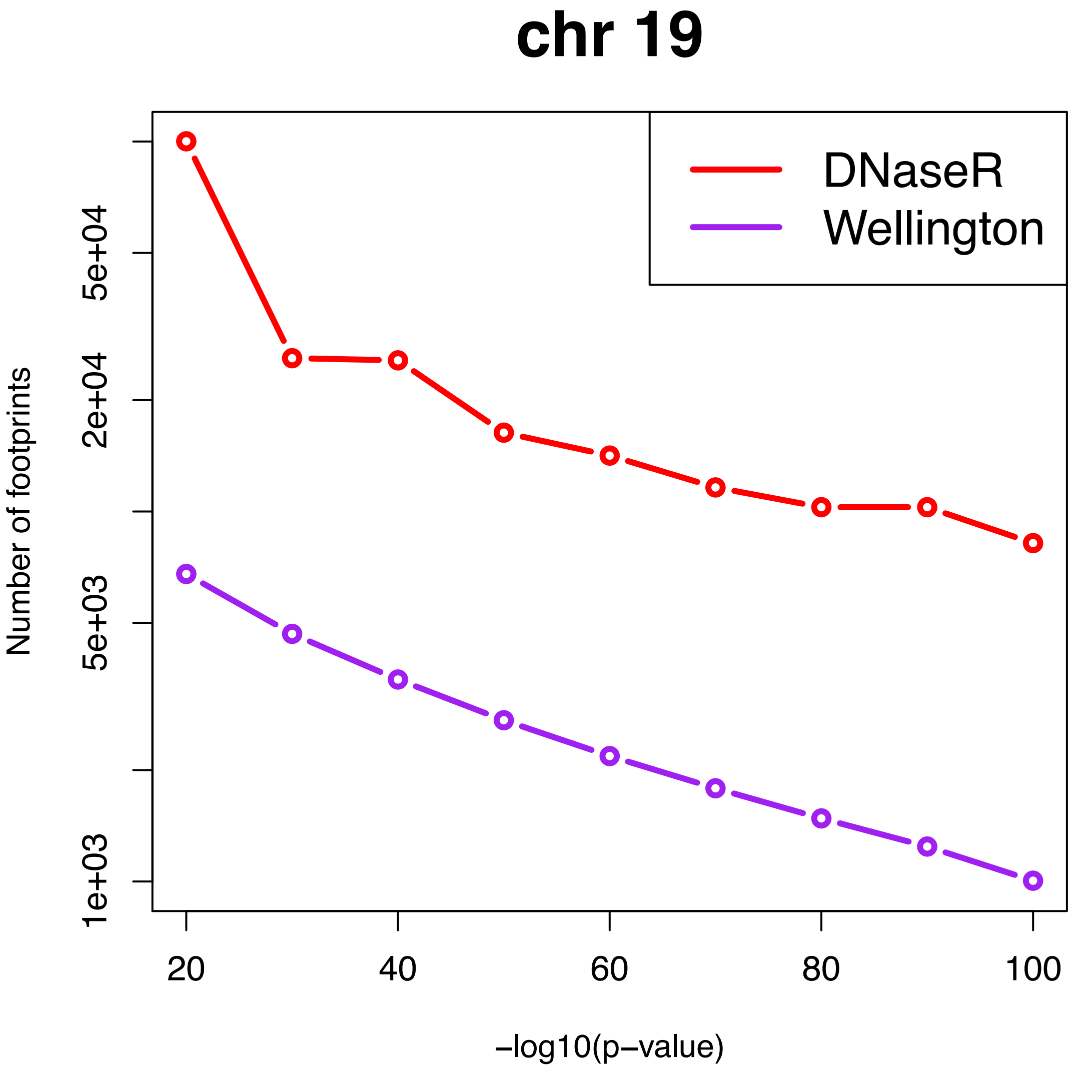

Supplementary Figure 1. Number of footprints obtained with different stringency thresholds.

Supplementary Figure 2

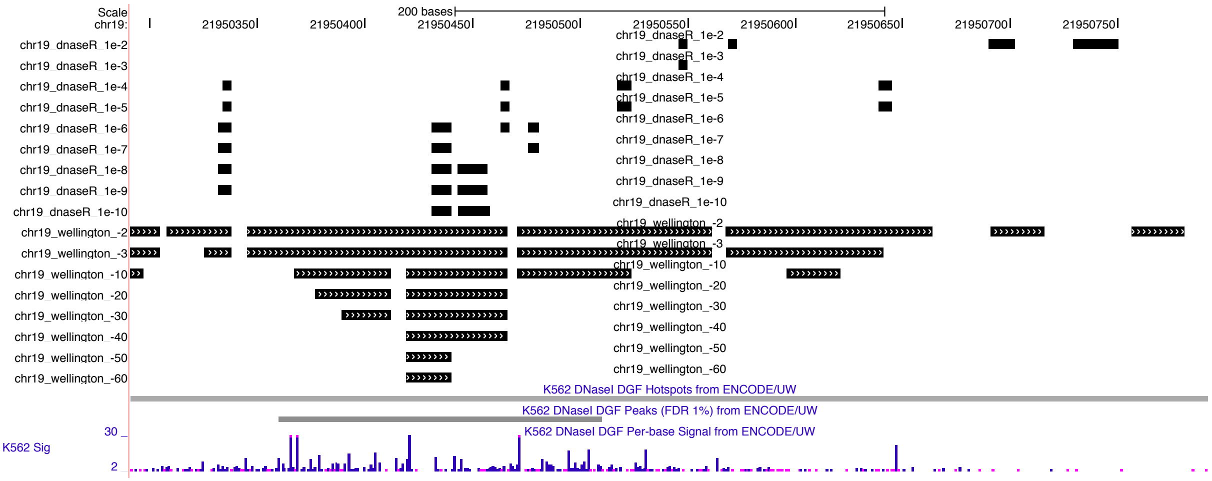

Supplementary Figure 2. Genome browser screenshot showing a genomic region along with footprints calls at different *p*-values (-10*log10(p)): when the stringency of the call is increased, Wellington maintains the strongest footprints, while DNaseR introduces new ones.

Supplementary Figure 3

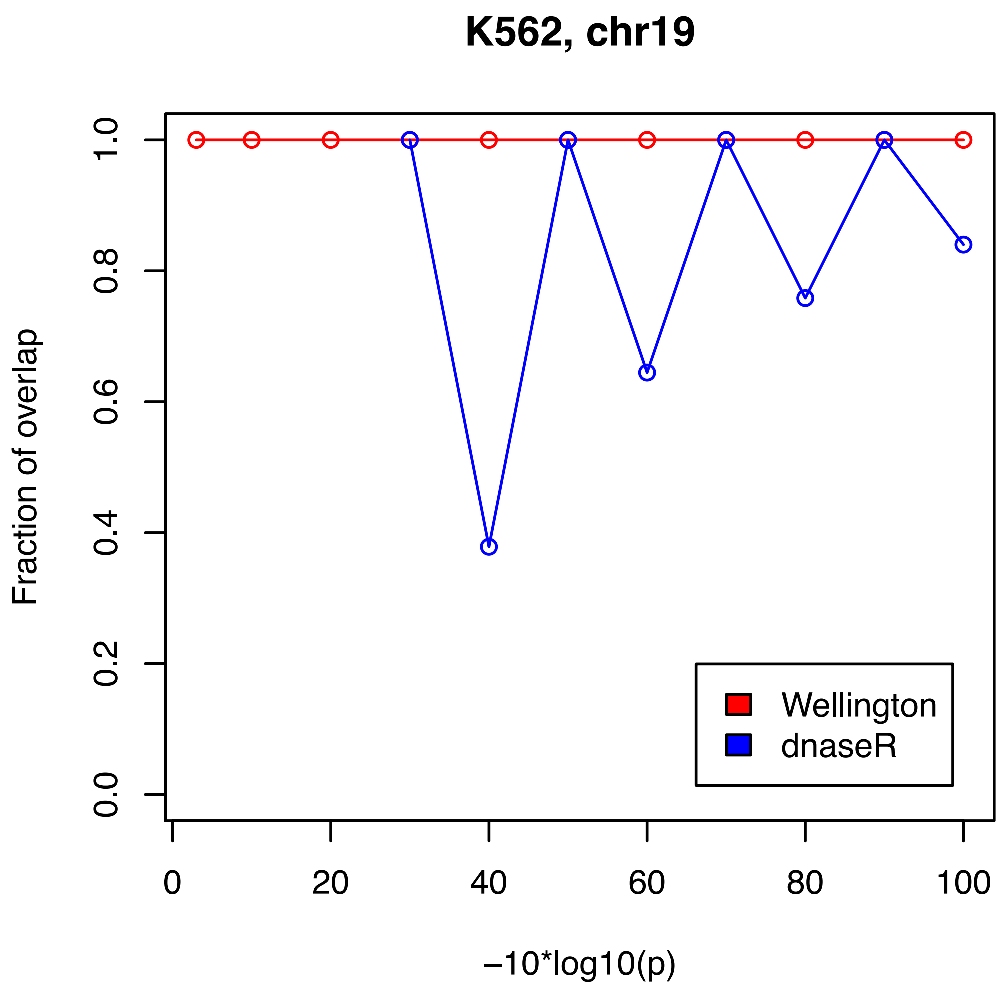

Supplementary Figure 3. : Each set of footprints has been compared to the set of footprints obtained at a lower *p*-value; the fraction of overlap is shown as a function of the *p*-value (-10*log10(p)).

Supplementary Figure 4

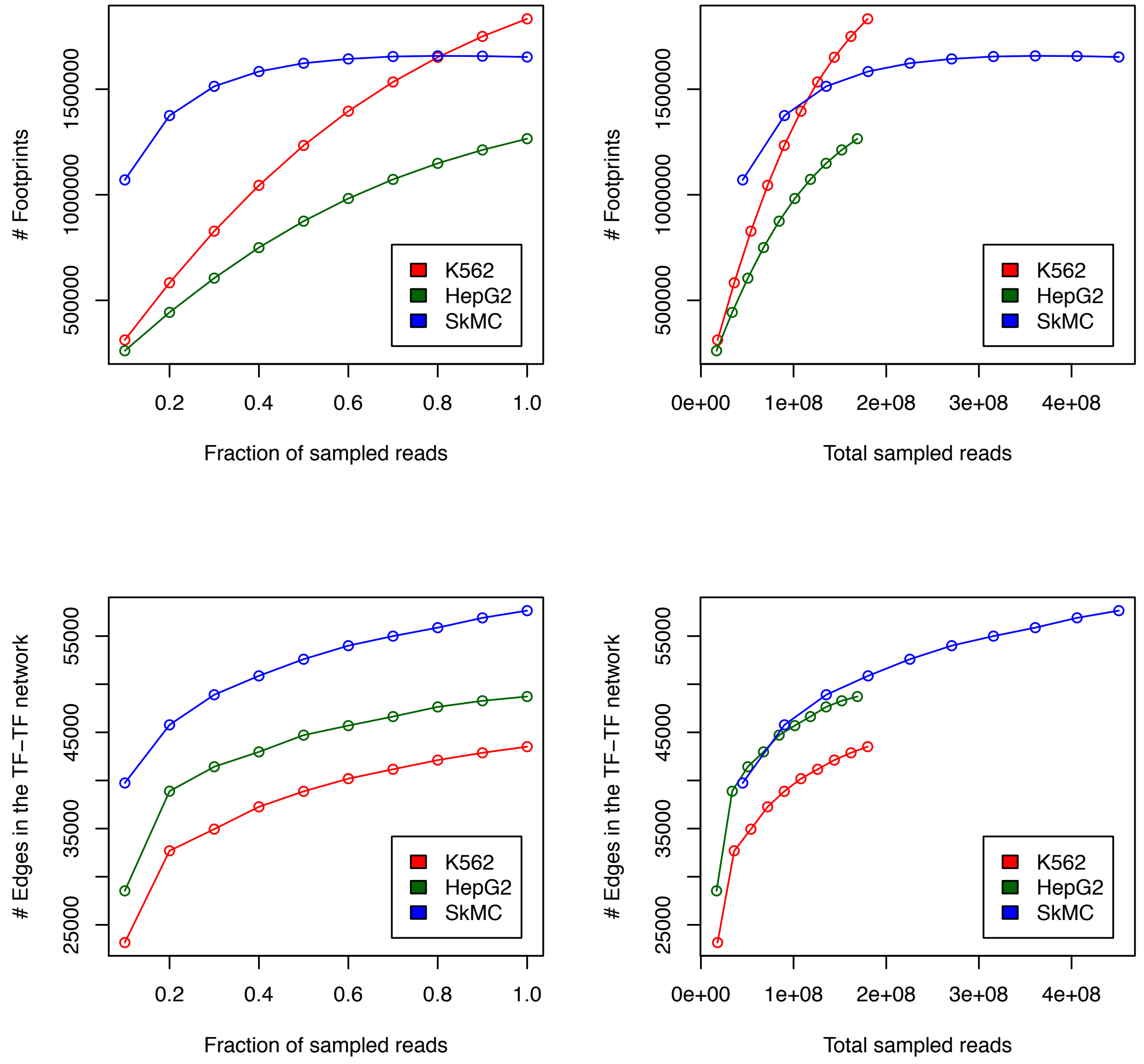

Supplementary Figure 4. : The total number of footprints and the number of footprints generating an edge in the TF-TF interaction network are shown as a function of the sampled reads.

Supplementary Figure 5

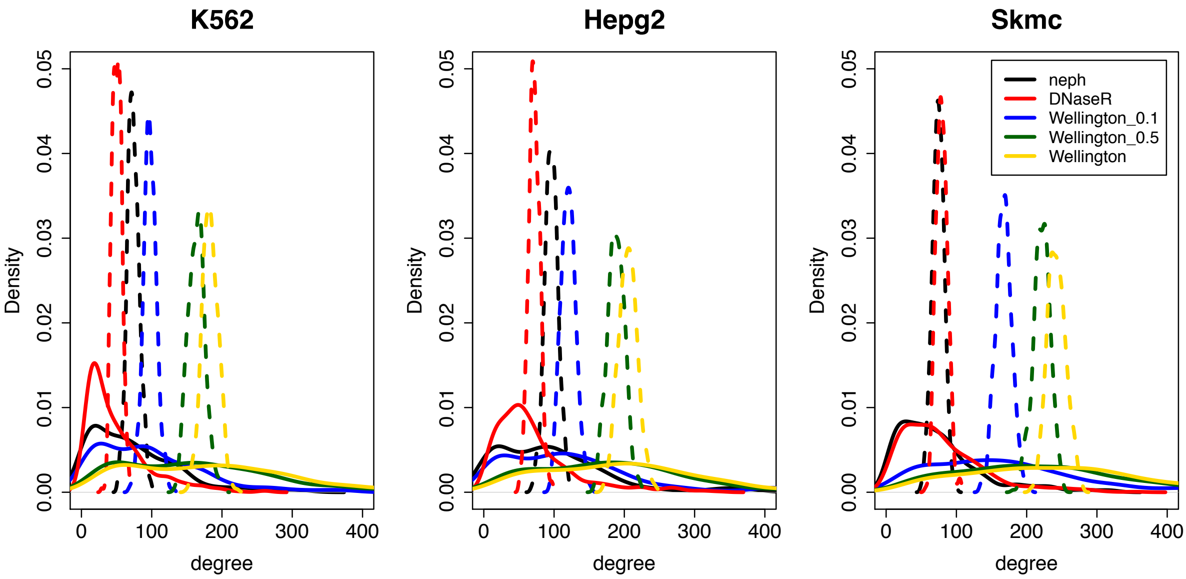

Supplementary Figure 5. : Degree distribution for TF-TF networks (solid lines) and corresponding random networks (dashed lines).

Supplementary Figure 6

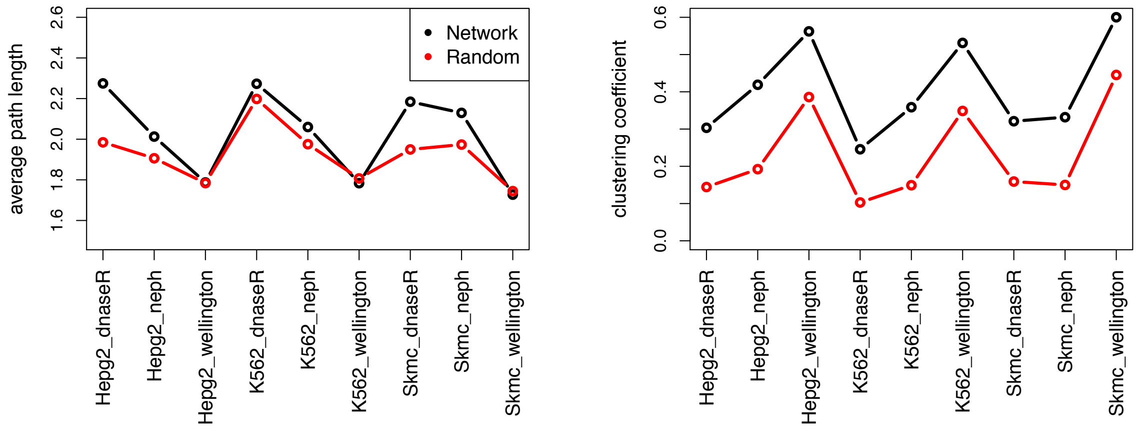

Supplementary Figure 6. : Average path length and clustering coefficient for TF-TF networks (black lines) and corresponding random networks (red lines).
